# Age-Associated Activation of the cGAS-STING Pathway and Impairment of DNA Damage Repair in Human Primary Alveolar Type II Cells

**DOI:** 10.14336/AD.2024.1175

**Published:** 2026-01-14

**Authors:** Chih-Ru Lin, Hassan Hayek, Hannah Simborio, Loukmane Karim, Karim Bahmed, Sudhir Bolla, Nathaniel Marchetti, Gerard J. Criner, Ying Tian, Beata Kosmider

**Affiliations:** ^1^Department of Biochemistry, School of Medicine, College of Medicine, Kaohsiung Medical University, Kaohsiung, Taiwan.; ^2^Center for Inflammation and Lung Research, Temple University, Philadelphia, PA 19140, USA.; ^3^Department of Microbiology, Immunology, and Inflammation, Temple University, Philadelphia, PA 19140, USA.; ^4^Department of Thoracic Medicine and Surgery, Temple University, Philadelphia, PA 19140, USA.; ^5^Aging & Cardiovascular Discovery Center, Temple University, Philadelphia, PA 19140, USA.; ^6^Department of Cardiovascular Sciences, Temple University, Philadelphia, PA 19140, USA

**Keywords:** alveolar type II cells, cigarette smoke, senescence, DNA damage, lung

## Abstract

Homeostatic imbalance and lung function decline are central physiological characteristics of aging and susceptibility to respiratory diseases. Senescence contributes to tissue damage and alveolar epithelial cell injury and decreases reparative capacity. Alveolar type II (ATII) cells have stem cell potential and self-renew to regenerate the alveoli after damage. They were isolated from younger and older non-smoker and smoker organ donors to define their function in the lung. Smoking and older age increased ATII cell senescence as detected by high β-galactosidase activity and P21 levels by Western blotting and RT-PCR. Also, the number of ATII cells was the lowest in lung tissue in older smokers. This was associated with increased stress signaling, as shown by elevated 4-HNE and G3BP1 expression in ATII cells, and inflammation indicated by high IL-8 levels in BAL fluid. In addition, DNA damage and decreased repair were observed using the comet assay, especially in ATII cells isolated from older smokers. This was accompanied by the highest levels of cytosolic double-strand DNA in this group and correlated with the activated cGAS-STING pathway and increased IRF3 expression. Moreover, telomere shortening, accumulation of TERRA molecules, and increased ZBP1 protein expression in ATII cells were associated with smoking and older age. Reduced NRF2 and DJ-1 expression in ATII cells was detected by Western blotting, especially in older smokers, which suggests an antioxidant defense system dysfunction. Our study provides insights into the impaired interconnected signaling network, which can contribute to ATII cell senescence.

## INTRODUCTION

Studies of healthy aging are critical from the clinical perspective since evidence indicates that various exogenous and endogenous factors can contribute to premature aging [1]. Also, the growing number of age-related lung diseases in limiting life and health spans highlights the importance of understanding this process [2]. There are structural and physiologic changes in aging characterized by decreased lung function. An impairment in tissue homeostasis is a central feature of aging [3]. Senescence has been linked to susceptibility to alveolar epithelial cell injury and decreased reparative capacity, contributing to cell dysfunction and damage. Alveolar type II (ATII) cells stabilize the alveolar structure and produce surfactant proteins that lower surface tension. These cells also drive the regenerative process of alveoli; they can self-renew and differentiate into alveolar type I cells after damage, indicating their critical role in the lung. Therefore, factors diminishing the stem cell capacity and function of ATII cells can contribute to lung aging.

Cigarette smoke contains chemicals, including acrolein, nicotine, and additives, and causes oxidative damage to biomolecules such as DNA, protein, and lipids [4, 5]. Evidence is accumulating in favor of the concept that stress signaling and inflammation, which are interdependent processes, are correlated with aging [6]. DNA oxidation can lead to various types of damage, including base modifications and loss and induction of DNA double-strand breaks (DSBs). DSBs induce DNA damage response (DDR) and rapidly initiate the phosphorylation of the histone H2A variant, H2AX, at serine 139 (γH2AX) [7-9]. The ataxia telangiectasia mutated (ATM) is the main kinase phosphorylating H2AX in response to DSBs generation, which causes the recruitment of repair machinery [10, 11]. Moreover, ATM is autophosphorylated immediately upon DSB formation [12]. γH2AX-foci accumulation has been reported in aging, and the ability of cells to repair damaged DNA can decline with age [13, 14]. DSBs are the most deleterious form of DNA damage, and if not repaired, they can contribute to cell senescence.

Recent evidence suggests that cell senescence is activated by the nucleic acids sensors cyclic GMP-AMP synthase (cGAS) and stimulator of interferon genes (STING) [15]. cGAS is induced by endogenous DNA of nuclear origin leaked into the cytoplasm [16]. Moreover, it activates DDR in a STING-dependent manner. STING is a key adaptor protein of the innate immune response to cytosolic DNA [17]. It partly resides in the nucleus and enhances DNA repair responses. These findings indicate the nuclear role of cGAS and STING in regulating genomic stability, which connects DNA damage to inflammation and cell senescence [18]. The activation of interferon regulatory factor 3 (IRF3) is controlled by the cGAS-STING pathway. IRF3 activity is required for the induction of genes encoding inflammatory cytokines [19]. Moreover, it has been suggested that Z-DNA binding protein 1 (ZBP1) may propagate the inflammatory signaling cascade specifically in response to telomere dysfunction [20].

There are gaps in our understanding of the molecular hallmarks of aging [2]. This process can impact ATII cells, and improving our knowledge is critical to strategies to counteract senescence and approaches to slow lung function decline in aging. Also, it can have implications for defining susceptibility to respiratory diseases. Here, we isolated ATII cells from younger and older non-smoker and smoker lung donors. We examined the relationship between DNA damage and the cGAS-STING pathway activation in the context of smoking and senescence. Also, the telomere’s function, which can affect nucleic acid-sensing machinery and innate immune signaling pathways, was analyzed in ATII cells. Our study provides novel insights into human primary ATII cell function in aging.

## MATERIALS AND METHODS

### Human ATII cell isolation

Human lungs were obtained from non-smoker and smoker organ donors whose lungs were unsuitable for transplantation and were donated for research through the Gift of Life Foundation (Philadelphia, PA), as we reported [21-23]. Donors were selected without a history of acute or chronic lung diseases with a PaO_2_/FIO_2_ ratio of >225 and an X-ray and clinical history not indicative of infection. Younger organ donors were 18-39 years old, and older were 60-82 years old. The Committee for the Protection of Human Subjects at Temple University approved this research.

ATII cells were isolated, and bronchoalveolar lavage (BAL) fluid was obtained, as we previously published [24]. Briefly, the lung was perfused and then instilled with 6.45 U/ml elastase (Roche Diagnostics, Indianapolis, IN). Lung tissue was minced, and the cell suspension was filtrated and purified by centrifugation on a density gradient made of Optiprep (Accurate Chemical Scientific Corp., Westbury, NY). A positive selection using Ep-CAM microbeads (Miltenyi Biotech, Germany) was applied to obtain ATII cells.

### Western blotting

ATII cells were lysed in a buffer with a protease and phosphatase inhibitor cocktail (Gold Biotechnology, Olivette, MO). Protein concentrations were quantified using BCA (Thermo Fisher Scientific, Waltham, MA). The cell lysates were then separated in polyacrylamide gradient gels (Thermo Fisher Scientific, Waltham, MA) and transferred to nitrocellulose membranes. The following antibodies were obtained from Santa Cruz Biotechnology (Dallas, TX) to determine protein expression by Western blotting: P21 (sc-397), DJ-1 (sc-55572), NRF2 (sc-365949), SIRT1 (sc-74504), and ZBP-1 (sc-271483). Antibody G3BP1 (13057-2-AP) was purchased from ProteinTech Group (Rosemont, IL). P-IRF3 (4947S) and IRF3 (4302S) were obtained from Cell Signaling (Danvers, MA). Additional antibodies were purchased: β-ACTIN (A5441; Sigma, St. Louis, MO), γH2AX (05-636; Millipore, Burlington, MA), 53BP1 (ab36823; Abcam, Cambridge, MA), LC3B (NB-100-2331; Novus Biologicals, Littleton, CO), and multiubiquitin (D058-3; MBL International, Schaumburg, IL). We used HRP-conjugated mouse or rabbit secondary antibodies (715-035-150 and 711-035-152; Jackson ImmunoResearch, West Grove, PA). The blots were developed using a chemiluminescent HRP detection reagent (WBLUF0500; Millipore, Burlington, MA) according to the manufacturer's instructions. NIH ImageJ software (Bethesda, MD) was used to quantify protein expression.

### RT-PCR

Total RNA was isolated using RNeasy Mini Kit (Qiagen, Hilden, Germany) and reverse-transcribed into cDNA by Superscript II Reverse Transcript kit (Thermo Fisher Scientific, Waltham, MA) according to the manufacturer’s instructions. PCR amplification was performed using the SYBR Green Master Mix kit (Thermo Fisher Scientific, Waltham, MA) and the StepOnePlus Real-Time PCR System (Applied Biosystems, Foster City, CA). The gene expression was analyzed by the ΔΔCt method. Telomere length was measured as described [25]. Applied primers are shown in Table 1.

**Table 1:** Primers used for RT-PCR.

Gene	Sequence (5’-3’)	
*36B4 for T/S*	d	CCC ATT CTA TCA TCA ACG GGT ACA A
	u	CAG CAA GTG GGA AGG TGT AAT CC
*53BP1*	Fwd	ATG GAC CCT ACT GGA AGT CAG
	Rev	TTT CTT TGT GCG TCT GGA GAT T
*cGAS*	Fwd	TAA CCC TGG CTT TGG AAT CAA AA
	Rev	TGG GTA CAA GGT AAA ATG GCT TT
*Chr 10q*	Fwd	GAA TCC TGC GCA CCG AGA T
	Rev	CTG CAC TTG AAC CCT GCA ATA C
*Chr 15q*	Fwd	CAG CGA GAT TCT CCC AAG CTA AG
	Rev	AAC CCT AAC CAC ATG AGC AAC G
*Chr Xq/Yq*	Fwd	GGA AAG CAA AAG CCC CTC TGA ATG
	Rev	ACC CTC ACC CTC ACC CTA AGC
*DJ-1*	Fwd	GTA GCC GTG ATG TGG TCA TTT
	Rev	CTG TGC GCC CAG ATT ACC T
*GAPDH*	Fwd	GGA GCG AGA TCC CTC CAA AAT
	Rev	GGC TGT TGT CAT ACT TCT CAT GG
*IRF3*	Fwd	AGA GGC TCG TGA TGG TCA AG
	Rev	AGG TCC ACA GTA TTC TCC AGG
*LC3B*	Fwd	AAG GCG CTT ACA GCT CAA TG
	Rev	CTG GGA GGC ATA GAC CAT GT
*NRF2*	Fwd	TCA GCG ACG GAA AGA GTA TGA
	Rev	CCA CTG GTT TCT GAC TGG ATG T
*P21*	Fwd	TGT CCG TCA GAA CCC ATG C
	Rev	AAA GTC GAA GTT CCA TCG CTC
*SIRT-1*	Fwd	TAG CCT TGT CAG ATA AGG AAG GA
	Rev	ACA GCT TCA CAG TCA ACT TTG T
*STING*	Fwd	CCA GAG CAC ACT CTC CGG TA
	Rev	CGC ATT TGG GAG GGA GTA GTA
*Tel for T/S*	1	GGT TTT TGA GGG TGA GGG TGA GGG TGA GGG TGA GGG T
	2	TCC CGA CTA TCC CTA TCC CTA TCC CTA TCC CTA TCC CTA
*ZBP1*	Fwd	AAC ATG CAG CTA CAA TTC CAG A
	Rev	AGT CTC GGT TCA CAT CTT TTG C

### ssDNA and dsDNA levels

QuantiFluor ssDNA and QuantiFluor dsDNA Systems (Promega, Madison, WI) were used to determine the levels of single-strand (ss) and double-strand (ds) DNA in BAL fluid according to the manufacturer’s instructions. A serial dilution of DNA standard was prepared and mixed with a fluorescent DNA-binding dye to obtain results using the SpectraMax fluorometer (Molecular Devices, San Jose, CA).

### Cell staining

A β-galactosidase staining kit (Cell Signaling Technology, Danvers, MA) was used to identify senescent cells. The staining was performed in freshly isolated ATII cells per the manufacturer’s instructions. Paraffin-embedded lung tissue sections were incubated with the following antibodies obtained from Santa Cruz Biotechnology (Dallas, TX): p-ATM (sc-47739), SP-C (sc-518029), dsDNA (sc-58749), TREX1 (sc-133112), 8-OHdG (sc-393871), cGAS (sc-515802) or purchased from other manufacturers: proSP-C (AB3786; Millipore, Burlington, MA), SP-A (NBP2-12928, Novus Biologicals, Littleton, CO), 4-HNE (MAB3249; R&D Systems, Minneapolis, MN), STING (PA523381; Invitrogen Corp., Carlsbad, CA). The corresponding secondary antibodies were obtained from Invitrogen Corp. (Carlsbad, CA): Alexa Fluor 594 (A21207, A21203), Alexa Fluor 488 (A21202), and Alexa 647 (A21447) and applied for 1h. Control staining was performed using IgG isotype antibodies. A fluoroshield mounting medium with DAPI (Abcam, Cambridge, MA) was used. Image analysis was conducted using a confocal laser-scanning microscope (Zeiss, Germany) and ImageJ software (NIH, Bethesda, MD).

### Comet assay

OxiSelect Comet Assay Kit (Cell Biolabs, Inc., San Diego, CA) was used to analyze DNA strand breaks in ATII cells, as we previously described [26]. ATII cells were also treated with 50μM etoposide (Cayman Chemical, Ann Arbor, MI) for 24h, followed by media removal to define repair capacity after 24h. Briefly, to analyze DNA damage, 1x10^5^ ATII cells were embedded in LMP agarose on a microscope slide, followed by incubation in a lysis buffer to denature DNA. An electrophoresis was performed using an alkaline condition in a horizontal chamber. The samples were dried, stained with a Vista Green DNA dye, and observed by fluorescence microscopy (Zeiss, Germany). The images were analyzed using OpenComet software, and the Olive tail moment was used to evaluate DNA damage [27].

### ELISA

IL-8 levels were measured in BAL fluid using ELISA based on standard techniques with slight modifications [28]. Briefly, recombinant human IL-8 standard (RP00052; Abclonal Technology, Woburn, MA) and an IL-8 antibody (bs-0780R; Bioss, Woburn, MA) were used. TMB Substrate (DY999; R&D Systems, Minneapolis, MN) was added for 30 min, and the reaction was stopped with 2N H_2_SO_4_. The absorbance was detected at 450 nm wavelength. The quantity of IL-8 was determined by comparing the optical density of the BAL samples to that in the standard curve comprised of various concentrations of the reference standard.

### Statistics

We evaluated the data for normality using the Anderson-Darling test. A Kruskal-Wallis test and one-way ANOVA were applied to determine statistically significant differences (*p*<0.05). Data are shown as means ± SEM from at least three independent experiments using different lungs. Relative data was normalized to younger non-smokers.

## RESULTS

### Oxidative stress in senescent ATII cells

**Figure 1.**
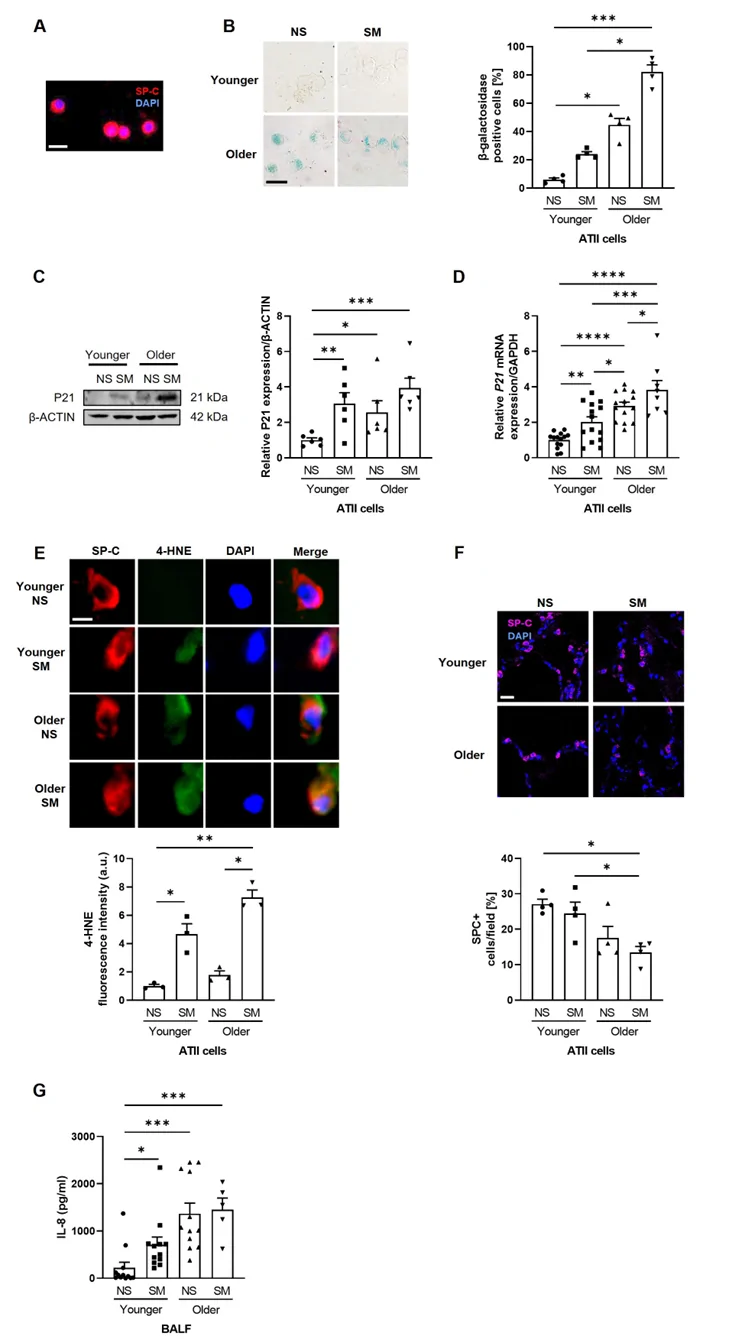
**ATII cell senescence, high oxidative stress, and pro-inflammatory response in older smokers**. ATII cells and BALF were obtained from younger and older non-smokers (NS) and smokers (SM). (**A**) The representative purity of isolated ATII cells using SP-C antibody by immunofluorescence (scale bar - 10 µm). (**B**) β-galactosidase assay using ATII cells (scale bar - 10 µm). (**C**) P21 expression in ATII cells by Western blotting. (**D**) *P21* mRNA levels were determined by RT-PCR. (**E**) Oxidative stress was analyzed using the 4-HNE antibody in ATII cells identified by SP-C staining in lung tissue sections (immuno-fluorescence, scale bar - 5 µm). (**F**) The percentage of ATII cells using SP-C antibody in lung tissue sections (immunofluorescence, scale bar - 50 µm). (**G**) IL-8 levels were analyzed in BALF by ELISA. Data are shown as means ± SEM. *p<0.05; **p<0.01; ***p<0.001; ****p<0.0001 (Kruskal-Wallis test in B, C, E, F, G, H and a one-way ANOVA in D).

Cellular senescence is one of the main characteristics of aged organisms [29]. We sought to determine how smoking and older age affect ATII cell function. ATII cells were isolated from non-smoker and smoker younger and older lung donors. The purity of freshly isolated ATII cells was examined using cell cytospins and SP-C staining by immunofluorescence (Fig. 1A). β-galactosidase assay was used to analyze ATII cell senescence. A significantly higher percentage of β-galactosidase-positive ATII cells was found in the older than younger non-smokers (Fig. 1B). Also, β-galactosidase activity was increased in ATII cells obtained from older smokers compared to younger lung donors. To analyze ATII cell senescence further, we determined P21 expression. Its levels were elevated in cells obtained from all analyzed groups compared to younger non-smokers, as detected by Western blotting (Fig. 1C). In addition, ATII cells isolated from older smokers had the highest *P21* mRNA levels as determined by RT-PCR (Fig. 1D). Its expression was also increased in older non-smokers than younger lung donors and in younger smokers compared to age-matched non-smokers. Our results suggest that P21 is activated by smoking and aging. Since we identified ATII cell senescence in older lung donors, we also wanted to determine the impact of smoking on oxidative stress and the pro-inflammatory response. We used 4-HNE as a marker of oxidative stress and found its elevated levels in ATII cells obtained from smokers compared to non-smokers by immunofluorescence (Fig. 1E). Also, 4-HNE expression was increased in older smokers than younger non-smokers. Moreover, ATII cells were examined using SP-C antibody by immunofluorescence. We found a lower percentage of ATII cells in older smokers than younger lung donors (Fig. 1F).

**Figure 2.**
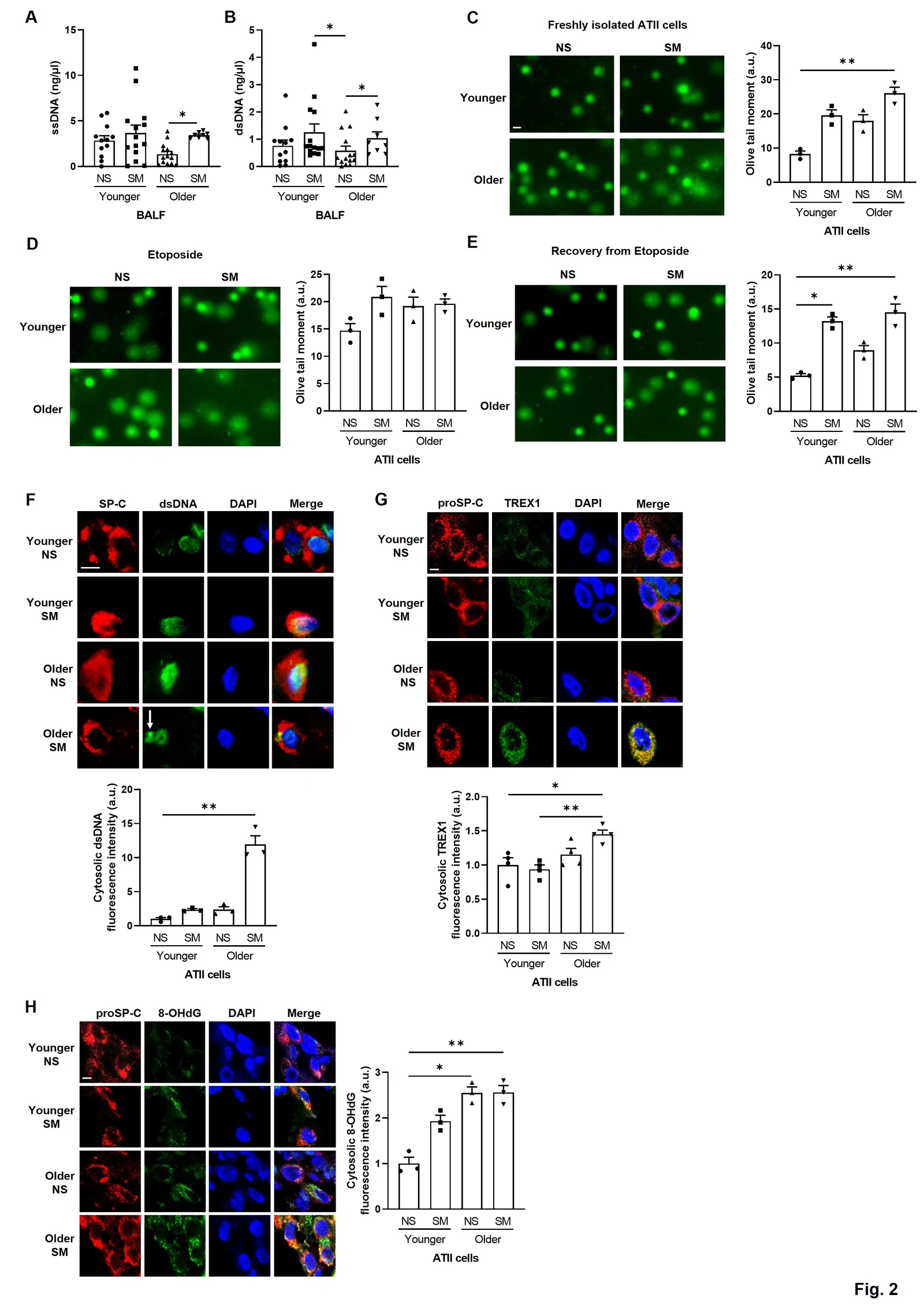
**Increased DNA damage, TREX1, and 8-OHdG levels in ATII cells in older smokers**. BALF, ATII cells, and lung tissue were obtained from younger and older non-smokers (NS) and smokers (SM). ssDNA (A) and dsDNA (B) levels in BALF. (**C**) DNA damage was analyzed in ATII cells by comet assay (scale bar - 5 µm). Quantification of the Olive tail moment is shown. (**D**) ATII cells were treated with etoposide for 24h to induce DNA damage. (**E**) Recovery was determined using drug-free media for 24h. (**F**) dsDNA cytosolic levels were analyzed in ATII cells identified using SP-C antibody (scale bar - 5 µm). Quantification of fluorescence intensity is also shown. (**G**) TREX1 expression in ATII cells was detected using a proSP-C antibody (scale bar - 5 µm). (**H**) 8-OHdG levels were analyzed in ATII cells identified using a proSP-C antibody (scale bar - 5 µm). Data are expressed as means±SEM.*p<0.05; **p<0.01 (Kruskal-Wallis test).

To assess a pro-inflammatory response in the lung, we examined IL-8 secretion in BAL fluid. Its levels were higher in older organ donors than in younger non-smokers, as detected by ELISA (Fig. 1G). Moreover, younger smokers had increased IL-8 expression than age-matched non-smokers. Together, these results indicate that smoking and aging increase oxidative stress and the pro-inflammatory response.

### Age-associated increased DNA damage and decreased repair capacity in ATII cells

We wanted to determine the impact of smoking and aging on DNA damage. Our results indicate elevated ssDNA and dsDNA levels in BAL fluid obtained from older smokers compared to age-matched non-smokers (Figs. 2A, 2B). Moreover, dsDNA levels were higher in younger smokers than older non-smokers. We further examined DNA damage in freshly isolated ATII cells from younger and older non-smokers and smokers by comet assay. Increased Olive tail moments were detected in ATII cells in older smokers than younger non-smokers (Fig. 2C). Next; we analyzed the DNA damage repair capacity in ATII cells. They were treated with etoposide for 24h to induce DNA damage (Fig. 2D). There was no significant difference in its levels in all the groups analyzed. ATII cells treated with etoposide were incubated in drug-free media for 24h, and the recovery from DNA damage was examined (Fig. 2E). We found a decreased DNA damage repair capacity in ATII cells obtained from younger and older smokers compared to younger non-smokers, as detected by increased Olive tail moments.

Since we found increased DNA damage, especially in older smokers in BAL fluid and ATII cells using comet assay, we wanted to determine cytosolic dsDNA in ATII cells. Our results indicate its highest levels in older smokers as detected by immunofluorescence (Fig. 2F). Next, we analyzed the TREX1 expression required for clearing cytosolic DNA. Its increased levels were detected in ATII cells obtained from older smokers compared to younger lung donors (Fig. 2G). To define the role of TREX1 further, we examined cytosolic 8-hydroxy-2-deoxy guanosine (8-OHdG) levels in ATII cells by immunofluorescence (Fig. 2H). Our results indicate its higher expression in older lung donors regardless of smoking status compared to younger non-smokers. Together, we found increased DNA damage, dsDNA accumulation in the cytosol, and decreased repair capacity, especially in older smokers.

### DNA damage signaling in ATII cells

**Figure 3.**
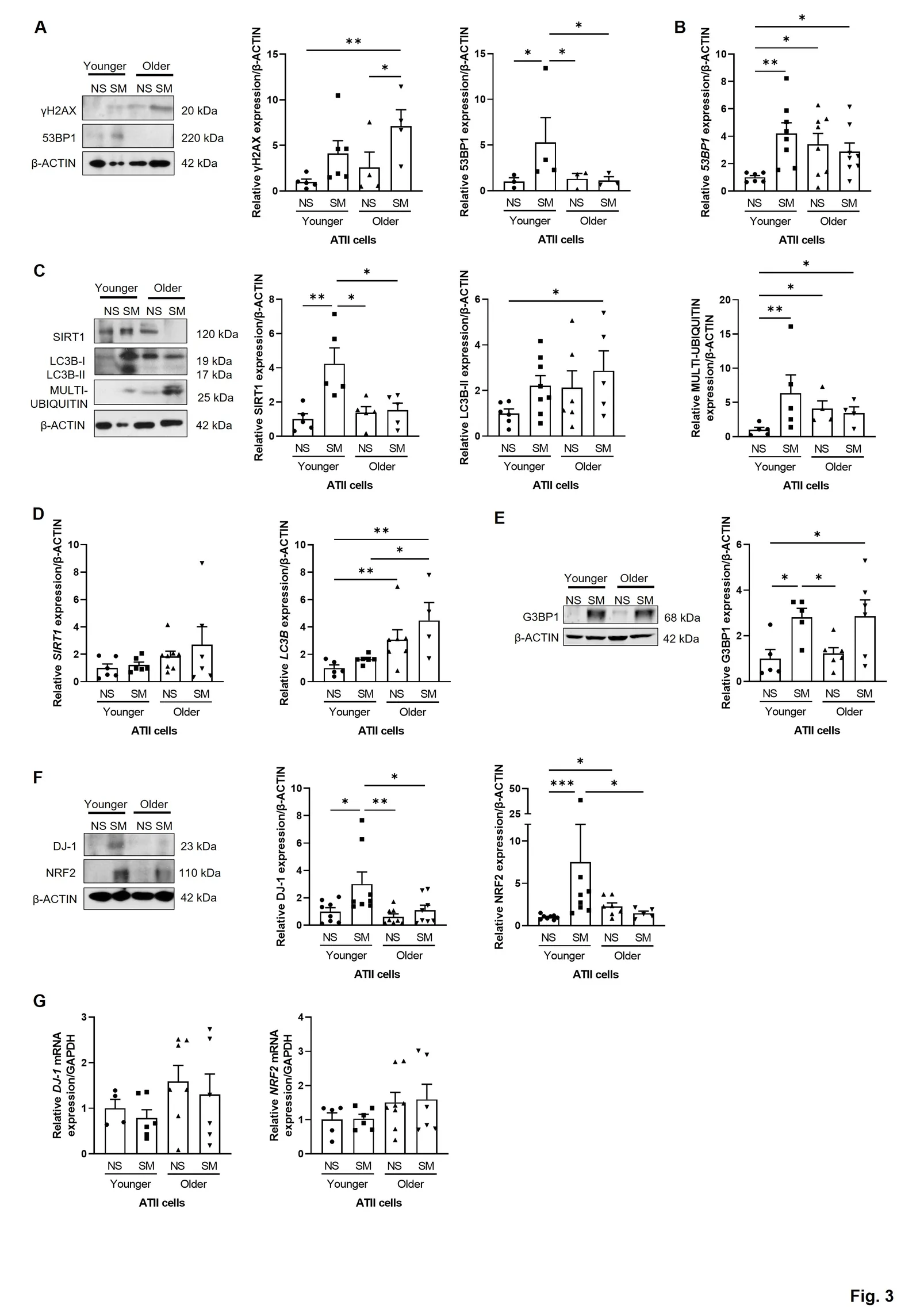
**Protein and gene expressions in ATII cells**. ATII cells were isolated from younger and older non-smokers (NS) and smokers (SM). (**A**) γH2AX levels and 53BP1 expression were analyzed in ATII cells by Western blotting. (**B**) *53BP1* levels were determined in ATII cells by RT-PCR. (**C**) SIRT1, LC3B-II, and multiubiquitin expression were analyzed in ATII cells by Western blotting. (**D**) *SIRT1* mRNA and *LC3B* mRNA levels were defined by RT-PCR. (**E**) G3BP1 expression was determined in ATII cells by Western blotting. (**F**) NRF2 and DJ-1 protein expression were analyzed in ATII cells by Western blotting. (**G**) *NRF2* mRNA and *DJ-1* mRNA levels were determined by RT-PCR. Data are expressed as means±SEM. *p<0.05; **p<0.01; ***p<0.001 (Kruskal-Wallis test).

Our results indicate smoking- and age-associated ATII cell dysfunction. Next, we wanted to determine DNA damage signaling by analyzing γH2AX levels, an early event in the DSBs damage response, and a central player in DDR [30]. As shown in Figure 3A, ATII cells isolated from older smokers had higher γH2AX levels than non-smokers, as detected by Western blotting. Moreover, 53BP1 protein expression, an important regulator of the cellular response to DSBs, was upregulated in younger smokers compared to non-smokers and older smokers. *53BP1* mRNA levels were the lowest in younger non-smokers compared to all analyzed groups using RT-PCR (Fig. 3B).

We further examined ATII cell function in younger and older non-smokers and smokers. Decreased SIRT1 levels were found in ATII cells isolated from older lung donors regardless of smoking status compared to younger smokers by Western blotting (Fig. 3C). Also, its expression was higher in younger smokers than in age-matched non-smokers. LC3B-II protein levels were increased in older smokers compared to younger non-smokers. Multiubiquitin expression was higher in all analyzed groups than in younger non-smokers, indicating the accumulation of ubiquitinated proteins. No significant differences were detected in *SIRT1* mRNA levels. Increased *LC3B* gene expression was observed in ATII cells in older smokers compared to younger lung donors (Fig. 3D). Also, ATII cells from older non-smokers had elevated *LC3B* levels than younger non-smokers. G3BP1 protein expression was higher in ATII cells in older smokers than younger non-smokers, as shown using Western blotting (Fig. 3E). Moreover, its levels increased in ATII cells in younger smokers than in non-smokers. Together, our results indicate age-associated ATII cell dysfunction, especially in older smokers.

### Downregulation of the antioxidant defense system in ATII cells in older adults

Activation of the antioxidant defense system counteracts oxidative stress induced by cigarette smoke [31]. Here, we wanted to determine the efficiency of this system in ATII cells. DJ-1 protein levels were the highest in ATII cells in younger smokers compared to all analyzed groups (Fig. 3F). NRF2 expression was increased in ATII cells in younger smokers than in age-matched non-smokers and older smokers, as detected by Western blotting. It was elevated in ATII cells isolated from older compared to younger non-smokers. However, we didn’t observe any significant changes in *DJ-1* and *NRF2* mRNA levels among all the groups analyzed by RT-PCR (Fig. 3G). Our results suggest smoking in ATII cells in younger adults activates the antioxidant defense system.

**Figure 4.**
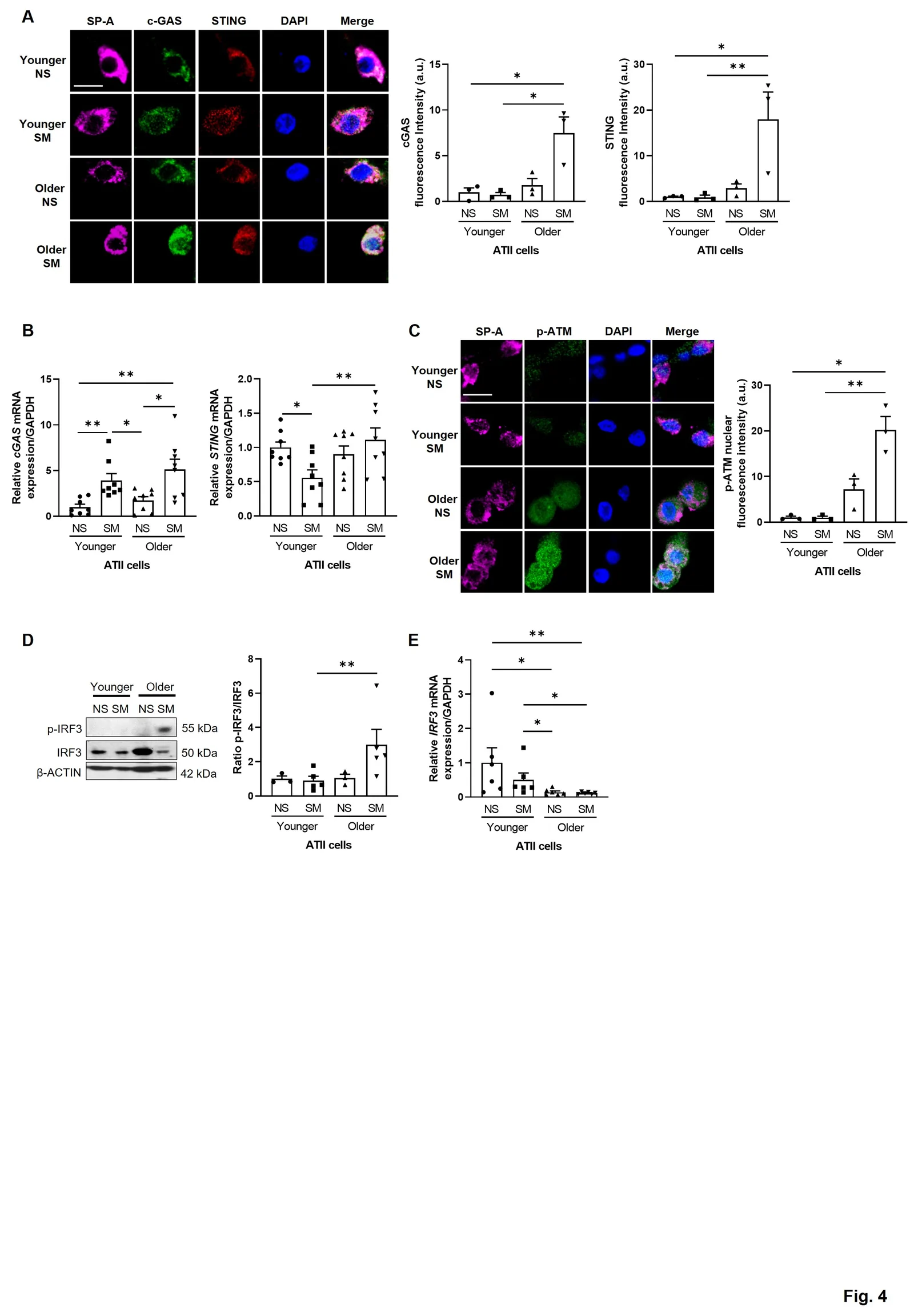
**Activation of the cGAS-STING pathway in ATII cells in older smokers**. Lungs and ATII cells were obtained from younger and older non-smokers (NS) and smokers (SM). (**A**) cGAS and STING expression were analyzed by immunofluorescence in ATII cells in lung tissue sections (scale bar - 10 µm). Quantification of fluorescence intensity is also shown. (**B**) *cGAS* and *STING* mRNA levels were determined by RT-PCR. (**C**) p-ATM expression was analyzed in ATII cells (immunofluorescence, scale bar - 10 µm). (**D**) p-IRF3 and IRF3 levels were analyzed in ATII cells by Western blotting. (**E**) *IRF3* expression was determined in ATII cells by RT-PCR. Data are expressed as means±SEM. *p<0.05; **p<0.01 (Kruskal-Wallis test in A, C, D, E, and one way ANOVA in B)

### Activation of the cGAS-STING pathway in ATII cells in older smokers

Although the cGAS-STING pathway is well known as a cytosolic DNA sensor, recent studies also indicate its critical role in response to DNA damage [17, 32, 33]. Moreover, STING partially localizes at the inner nuclear membrane; therefore, we wanted to examine the association of this signaling in ATII cells in aging and smoking. We detected the highest cytosolic dsDNA levels in ATII cells in older smokers (Fig. 2F) and wanted to determine whether DNA leak could activate this signaling. cGAS and STING levels were analyzed in ATII cells using immunofluorescence and RT-PCR. Higher cGAS expression was detected in ATII cells isolated from older smokers compared to younger lung donors (Fig. 4A). Notably, STING levels correlated with cGAS expression.

*cGAS* and *STING* mRNA expression was analyzed in ATII cells. *cGAS* levels were increased in ATII cells obtained from smokers than non-smokers regardless of age (Fig. 4B). *STING* mRNA expression was elevated in ATII cells obtained from older smokers than younger smokers.

ATM is activated by autophosphorylation on Ser1981 in response to DSBs [34]. We considered the possibility that ATM is involved in the cGAS-mediated response. Our data indicate that p-ATM levels were higher in ATII cells in older smokers than younger lung donors (Fig. 4C). This correlated with elevated cytosolic dsDNA accumulation and upregulated cGAS and STING expression, suggesting ATM activation.

The downstream effectors of pATM and the cGAS-STING pathway were explored further in ATII cells. IRF3 phosphorylation at serine 396 is a marker of the STING pathway activation [35]. Our results show increased pIRF3 levels in ATII cells isolated from older than younger smokers (Fig. 4D). ATII cells obtained from older lung donors had lower IRF3 mRNA expression compared to younger ones (Fig. 4E). Together, our findings suggest the activation of the cGAS-STING pathway at the posttranslational level in ATII cells in older smokers, which was associated with DNA damage and senescence.

**Figure 5.**
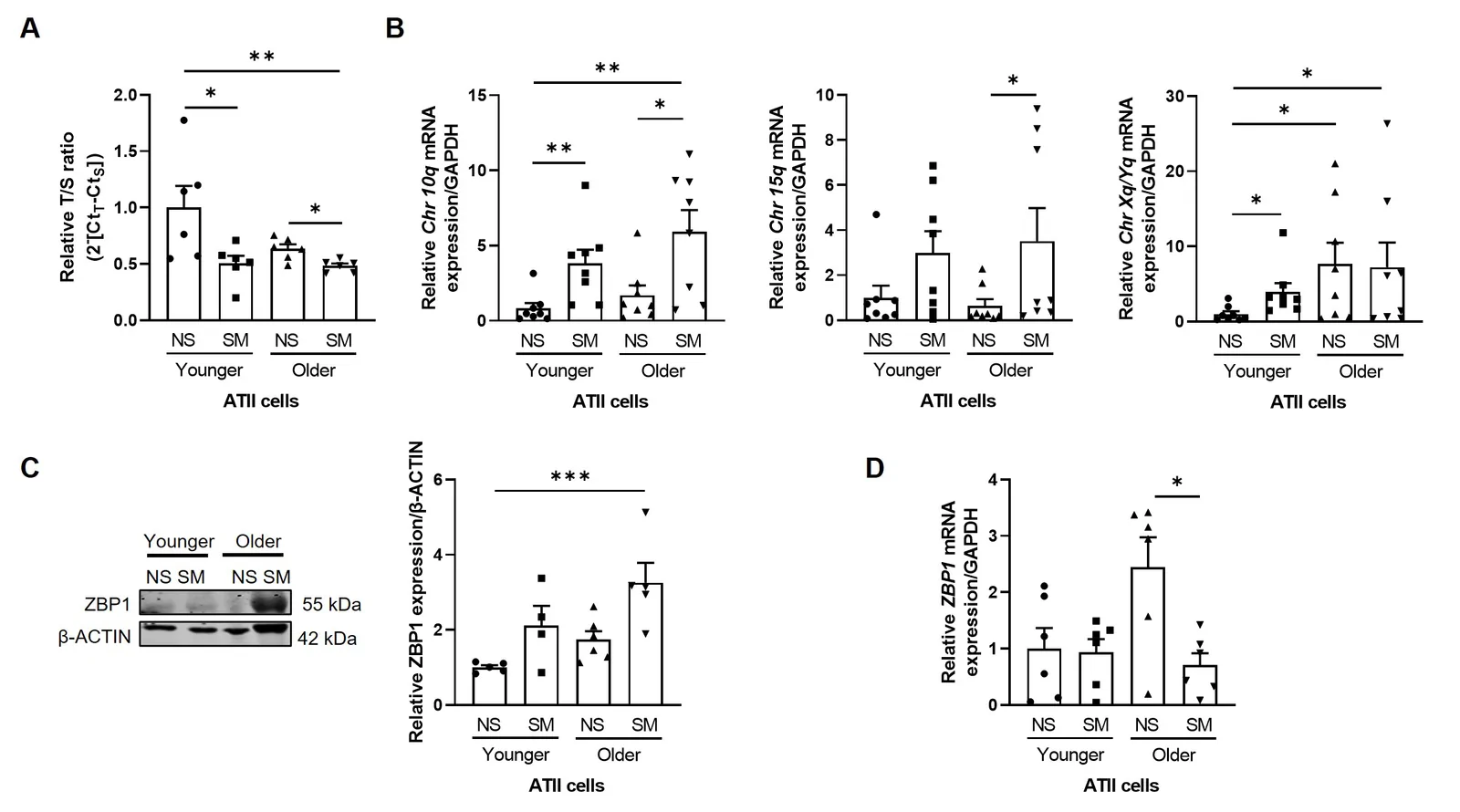
**Telomere shortening and increased TERRA levels in ATII cells in older smokers**. ATII cells were isolated from younger and older non-smokers (NS) and smokers (SM). (**A**) Telomere length measurement by qPCR. (**B**) TERRA transcript levels from chromosomes 10q, 15q, and Xq/Yq were determined in ATII cells by RT-PCR. (**C**) ZBP1 protein expression was analyzed in ATII cells by Western blotting. Densitometric quantification is also shown. (**D**) *ZBP1* mRNA levels were analyzed by RT-PCR. Data are expressed as means±SEM. *p<0.05; **p<0.01; ***p<0.001 (Kruskal-Wallis test).

### Dysfunctional telomeres and increased TERRA accumulation mediate ZBP1 activation

Telomere dysfunction and shortening can contribute to lung aging [36]. We analyzed the telomere lengths in ATII cells. They decreased in older smokers than younger non-smokers and in smokers compared to age-matched non-smokers (Fig. 5A). These results indicate age- and smoking-associated telomere length alterations in ATII cells. Dysfunctional telomeres are transcribed into long non-coding RNA species termed TERRA (telomeric-repeat-containing RNA) [37, 38]. We assessed the TERRA transcript levels from chromosomes 10q, 15q, and Xq/Yq by RT-PCR. The analysis revealed higher TERRA transcripts from chromosome 10q in ATII cells obtained from smokers compared to age-matched non-smokers and in older smokers than younger non-smokers (Fig. 5B). Its levels from chromosome 15q were increased in ATII cells in older smokers than non-smokers. TERRA transcripts from chromosomes Xq/Yq were elevated in all analyzed groups compared to younger non-smokers. These results indicate aging and smoking-associated telomere dysfunction and the accumulation of TERRA molecules.

It has been reported that the innate immune sensor, ZBP1, can also sense RNAs that might exist in the Z conformation [20]. Moreover, ZBP1 binds TERRA. Since we detected increased TERRA levels, especially in ATII cells isolated from older smokers, we wanted to analyze ZBP1 expression. ZBP1 protein levels were upregulated in older smokers compared to younger non-smokers (Fig. 5C). *ZBP1* mRNA levels were significantly higher in ATII cells obtained from older non-smokers compared to age-matched smokers (Fig. 5D). Together, our results indicate that telomere dysfunction associated with aging and smoking contributes to the accumulation of TERRA molecules and high ZBP1 protein expression in ATII cells.

**Figure 6.**
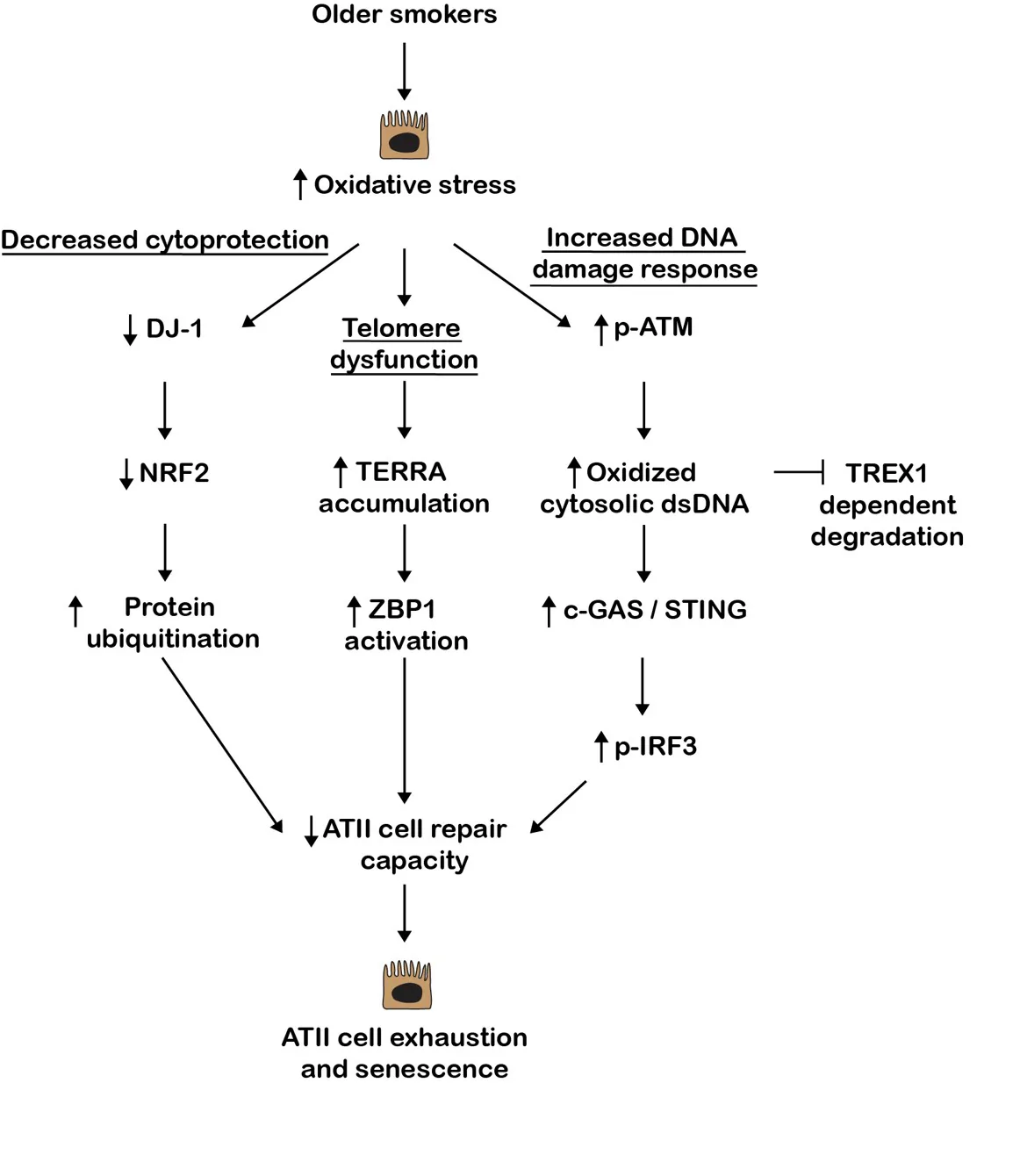
**The model of dysregulated pathways in ATII cells associated with aging and smoking**. ATII cells in older smokers have increased oxidative stress, impaired antioxidant defense system, telomere dysfunction, and dysregulated DNA damage response. This leads to decreased ATII cell repair capacity and exhaustion.

## DISCUSSION

Lung aging is associated with pulmonary function decline due to oxidative stress and inflammation, followed by structural alveolar changes [39]. Cigarette smoke has been suggested to contribute to cellular senescence. The novelty of this study is using ATII cells isolated from younger and older organ donors to define the interconnection between smoking and aging. We identified dysregulated factors that drive ATII cell senescence. Our findings can have implications for age-related diseases and treatment strategies.

Cigarette smoke can induce oxidative stress, leading to protein, nucleic acids, and lipid damage and cause inflammation [5, 40]. We have previously shown higher oxidative stress in primary ATII cells isolated from heavy smokers, highlighting the imbalance between reactive oxygen species (ROS) and the antioxidant defense system [41]. Here, elevated 4-HNE levels were detected in ATII cells in smokers regardless of age compared to non-smokers. Stress granules are condensates of non-translating messenger ribonucleoproteins (mRNPs) originating from mRNAs stalled in translation initiation via a network of interactions involving the RNA-binding protein G3BP1 [42]. We detected increased G3BP1 levels in smokers, which indicates stress conditions. Studies have shown that stress granule response can be used to assess the toxicity of various factors in the lung [43]. Also, cigarette smoking can alter the pro-inflammatory responses and increase macrophage recruitment and activation to release inflammatory mediators [44]. We observed elevated IL-8 levels in BAL fluid in all analyzed groups compared to younger non-smokers. Our previous report showed high IL-8 levels in ATII cells isolated from smokers, although the impact of aging has not been studied [41]. Furthermore, increased oxidative stress and pro-inflammatory responses induced by cigarette smoke are linked to DNA damage [45]. Our results indicate increased ssDNA and dsDNA levels in BAL fluid from older smokers. Also, younger smokers had higher dsDNA levels. We reported elevated DNA damage in ATII cells in smokers compared to non-smokers; however, different age groups were not analyzed [26]. Moreover, ATII cells isolated from younger and older non-smokers and smokers were analyzed by comet assay. We examined the ability of ATII cells to repair DNA damage induced by etoposide. Increased Olive tail moments were detected in ATII cells in smokers regardless of age, which indicated their decreased repair capacity. We also found the highest levels of cytosolic dsDNA in ATII cells in older smokers, which suggests its leak, accumulation, and impaired DNA repair.

To further determine the mechanism of ineffective DNA damage repair, we analyzed TREX1 levels, the major cytoplasmic 3′-5′ exonuclease in mammalian cells essential for this process [46]. Interestingly, we found its elevated expression in ATII cells in older smokers than in younger lung donors. To further define TREX1 dysfunction in removing cytosolic dsDNA, 8-OHdG levels were analyzed. It is as a marker of oxidative DNA damage and a factor that plays a role in many pathophysiological processes and aging [47]. We found increased 8-OHdG expression in ATII cells in older lung donors. It is worth noting that oxidized DNA modified with 8-OHdG is resistant to cytosolic exonuclease TREX-1-mediated degradation [48]. This may explain elevated DNA damage and 8-OHdG levels despite higher TREX1 expression in this group, which contributes to impaired DNA damage repair in older smokers.

During normal adult homeostasis, the lung is quiescent, exhibiting a low cellular turnover rate [49]. Quiescence also helps to prevent replication-associated DNA damage. Cell injury reactivates quiescent cells to proliferate for lung repair and regeneration [50]. However, aberrant niche activities compromise cell functions and contribute to tissue aging and disease pathogenesis. Indeed, replicative stress has been implicated as a 'driver' of aging in different cell types [51]. Downregulation of DDR and repair proteins can cause accumulation of DNA damage, leading to dysfunction of cell self-renewing populations. γH2AX is a central player in the DDR, a network of cellular pathways that sense, signal, and repair DSBs [7, 8]. Its increased levels were observed in ATII cells isolated from older smokers. However, DNA damage analysis using comet assay indicates lower repair capacity in this group, suggesting that DDR signaling failed to activate DNA damage repair. Also, 53BP1 is involved in the DDR [17, 52]. Decreased 53BP1 protein expression in ATII cells in older smokers indicates impaired DNA damage repair. This correlated with high oxidative stress and pro-inflammatory responses.

Cells lacking efficient DNA repair mechanisms can accumulate DSBs and release dsDNA into the cytosol [53]. Failure to degrade dsDNA by TREX triggers the cGAS-STING pathway. Moreover, cGAS-STING signaling activation has been linked to genome stability [54-56]. Notably, it has been shown that oxidatively modified DNA, e.g., 8-OHdG, can still activate cGAS [57]. We found the highest dsDNA in the cytosol and cGAS and STING protein expression in ATII cells isolated from older smokers. Recently, it has been shown that DNA release and cGAS-STING pathway activation can drive lung disease [58]. Also, it was found that STING is required for cGAS-mediated senescence by activating IRF3 to upregulate the secretory-associated-senescence phenotype (SASP) [59]. Our results suggest that the cGAS-STING-IRF3 axis is activated by cigarette smoke-induced DNA damage in ATII cells in older smokers and regulated at the posttranslational rather than transcriptional level. This provides novel insights into the association between this pathway and smoking in the context of ATII cell senescence.

SIRT1 is an anti-aging protein that regulates oxidative stress and enhances DNA damage repair capacity [60, 61]. The loss of SIRT1 is indicative of cell cycle arrest and cellular senescence [39]. It has been reported that SIRT1 expression was decreased in human ATII cells treated with cigarette smoke media suspension [62]. Moreover, its pharmacological activation by SRT2104 significantly reduced ATII cell senescence induced by this treatment. Here, we found decreased SIRT1 levels in ATII cells obtained from older lung donors and increased expression in younger smokers. This may indicate the activation of SIRT1-mediated cytoprotective mechanisms in the latter group. Our results suggest altered age-related responses in ATII cells.

Chen et al. [63] showed that HBE and SAE cell lines exposed to cigarette smoke had high LC3B-II levels, which is a marker for autophagosomes. Also, it was reported that alveolar macrophages obtained from smokers or exposed to cigarette smoke extract had increased LC3B-II levels [64]. This indicates a block in autophagic flux rather than improved functioning of the autophagosome generation machinery. Our results indicate increased LC3B-II expression in ATII cells in older smokers, suggesting their dysfunction.

It has been shown that cigarette smoke and aging modulate proteostasis, contributing to the accumulation of ubiquitinated proteins, pro-inflammatory responses, and oxidative stress [65]. We found significantly increased multiubiquitin expression in ATII cells isolated from all analyzed groups compared to younger smokers. Since we detected high IL-8 and 4-HNE levels in ATII cells induced by cigarette smoking and aging, we wanted to define the efficiency of the antioxidant system. It has been reported that lung aging is associated with a progressive decline in antioxidant defense capacity [66, 67]. Also, the ability to prevent oxidative damage in cells and the potential to regenerate lung tissue are two key determinants of this process [6]. Continuous exposure to oxidative stress induced by cigarette smoke triggers a defense system. The transcription factor NF-E2-related factor 2 (NRF2) is a key regulator of the cellular antioxidant response [68]. It has been shown to orchestrate the first line of defense against oxidative insults to damaging factors and pro-inflammatory cytokines secreted by senescent cells. However, its function in the context of smoking and human primary ATII cell senescence is not well known. DJ-1 protects against ROS and modulates NRF2-mediated functions [41]. We found increased NRF2 and DJ-1 levels in younger smokers, which suggests antioxidant defense system activation. However, their expression was decreased in older smokers, indicating impaired cytoprotective signaling. Studies showed that targeting the NRF2 pathway is critical in alleviating age-related structural alterations in lung tissue and functional decline [69].

DNA damage can activate ATM, which is a serine/threonine kinase that responds to DSBs and controls DDR [70]. We showed increased p-ATM levels in ATII cells in older smokers compared to younger lung donors. This suggests the enhanced sensitivity of ATII cells to cigarette smoke-induced DNA damage associated with senescence. The recognition and elimination of senescent cells can increase the proliferative capacity of stem cells and enhance tissue repair [71, 72]. Studies also indicated that p21 knockout supports tissue regeneration [73-76]. Therefore, inhibiting P21 to eliminate senescent cells may be a promising strategy for age-related lung diseases associated with smoking.

Furthermore, our results indicate that increased p-ATM levels correlated with the accumulation of cytosolic dsDNA and pIRF3 protein upregulation in ATII cells in older smokers. Also, ATII cells isolated from this group had elevated TERRA levels and the highest ZBP1 protein expression, suggesting telomere dysfunction. It was reported that ZBP1 senses TERRA-containing transcripts, and their interaction was shown [20]. Our results suggest cytosolic dsDNA accumulation and dysfunctional telomeres marked by elevated TERRA contribute to a ZBP1-dependent response in ATII cells in older smokers. Together, our data point to new insights into how smoking in older adults may lead to the impairment of the DDR, contributing to ATII cell senescence (Fig. 6). We show the interconnected network, including the shortening of telomeres, increased TERRA and pIRF3 levels. This is also associated with higher ZBP1 protein expression, which can contribute to accelerated ATII cell senescence, especially in older smokers. In addition, the cGAS-STING pathway was activated, and our data suggest that it may serve as a potential target in lung diseases associated with smoking.
